# Subperiosteal Resection of a Rib for Empyema in Children—Recovery

**Published:** 1888-02

**Authors:** Albert B. Strong

**Affiliations:** Chicago, Ill.


### SUBPERIOSTEAL RESECTION OF A RIB FOR EMPYEMA IN CHILDREN—RECOVERY.*

BY ALBERT B. STRONG, M. D., CHICAGO, ILL.

I have within the past two years made subperiosteal resection of a segment of a rib for empyema in two young persons, one a boy of six and the other a youth of seventeen years of age. The results have been so satisfactory that I desire to place the cases on record at the present time, particularly so since able authority has recently condemned the operation in children.[a]

*Case 1.*—In May, 1886, I saw in consultation with Dr. Charles Venn, of this city, a boy six years of age. The father, mother and other children of the family were perfect pictures of health. The boy was large for his age, and up to his present illness had always enjoyed the best of health. Four weeks prior he began to cough, and eight or nine days later evidences of an effusion into the right pleural cavity were manifest. In spite of appropriate constitutional and local treatment, the effusion increased, and when I saw him the chest cavity was completely filled. The boy was much emaciated with continued fever and night-sweats.

A hypodermic needle thrust into the chest just above the eighth rib in the axillary line revealed the presence of pus. Under ether the eighth rib was exposed through a parallel incision two inches in length. The periosteum, midway between the upper and lower border of the rib, was split in a longitudinal direction the distance of an inch. By means of a blunt hook the periosteum and intercostal vessels were easily pealed off from the posterior surface of the rib, and three-fourths of an inch was cut away with bone-nippers. This was done without wounding the intercostal vessels or pleura. Next the internal layer of the

*Read before the Chicago Medical Society October 17, 1887.

[a]See an article by Dr. Garnett, of Washington, on "The Surgical Treatment of Suppurative Pleuritis in Children."

periosteum was split to the same extent and parallel to the external layer. The pleura was opened when two quarts of laudable pus were evacuated. The chest cavity was immediately washed out with a warm saturated solution of boracic acid, and a large rubber drainage tube retained in position.

The subsequent history was uneventful. He began to improve at once. The chest cavity was daily washed out with simple warm water. A wad of oakum was placed over the wound. The tube was kept in place six weeks, when the lung was entirely expanded and the wound healed completely. Now, sixteen months after the operation, the boy is perfectly well. The right chest is normal, like its fellow, in shape and function. There is but a small scar at the seat of operation, and the rib has perfectly re-formed in direction and shape.

*Case 2.*—Norwegian boy, seventeen years of age, always delicate, very thin and poorly nourished, six feet tall, pale and anæmic. Father, brother and one sister died of consumption. Mother alive and doing well. Patient was first taken sick about March 10, 1887. The symptoms and signs indicated pleural pneumonia of the lower lobe of the right lung. In a few days he was convalescent.

On March 24 he had a relapse, and the right chest slowly and intermittingly filled with effusion. He had night-sweats, diarrhœa and cough, and a temperature of 102 to 104 degrees F. On April 24, about five weeks from the commencement of the effusion, empyema being diagnosed by the hypodermic syringe, with the assistance of Dr. Venn, I removed subperiosteal, one and one-half inches of the ninth rib in the axillary line. Four quarts of laudable pus escaped. At the last about four ounces of clear serum passed out. Immediately washed out the chest cavity with a hot solution of boracic acid, introduced two large parallel rubber tubes with a drainage diameter of three-eighths of an inch each. These were held in position by a rubber shield and elastic bandage around the chest. The pleura was felt to be about three-fourths of an inch thick. During the next few days there were washed out large masses of lymph. Hot water injections of two quarts at a time were used, night and morning.

A wad of oakum was kept over the opening and changed often. The diarrhœa, sweating and cough began immediately to abate. The temperature in three days dropped to normal. On the first day of May, eight days after the operation, the tubes were crowded out. All the unfavorable symptoms had disappeared; he was eating and sleeping well. The opening on the inside was closed, as I then thought by the expansion of the lungs. I subsequently learned it was closed by the arching of the diaphragm. The tube was left out five days, when the temperature began to ascend and soon reached 104. There was a return of the sweating, diarrhœa and cough. Nothing had escaped from the wound in the meantime. I then re-opened the wound and washed out about five ounces of stinking pus and debris, passed a large Van Buren urethral sound into the chest, and ascertained that the lung was compressed into a small wad against the vertebral column. It had not expanded a particle. I felt the apex of the sound in the chest above the clavicle. Re-introduced longer, fenestrated drainage tubes three inches in length. From this time on his convalesence was uninterrupted; temperature quickly became normal and did not again rise. He soon began to go out of doors and improved rapidly. On June 12, three weeks after the tubes were re-introduced, my notes read: Boy has gained ten pounds; out of doors most all day; eats enormously and digests it well; no cough and but little discharge. The sound in the chest shows the lung has not expanded. Put in drainage tubes without fenestra, since granulations had crowded into the former tubes so as to nearly occlude them. Sent the boy to a farm near Whitewater, Wisconsin, with instructions to keep tubes in position and syringe out the cavity once a day with warm water that had been boiled. Ten weeks later, on August 25, he returned, looking altogether different; was twenty pounds heavier than when first taken sick, and expressed himself as never feeling better in his life. The tubes had remained in place until a few days before, when they had been crowded out and could not be re-introduced. Physical examination showed the lung expanded with good motion of the chest and vesicular murmur throughout. In a few days more the opening entirely healed and the boy left for Norway perfectly recovered—six months from the commencement of his illness and four months wearing the tube.

It is only within a few years that surgeons have been bold enough to make a section of a rib for empyema. It is perhaps but natural that the operation should be condemned by those who have always used the trocar or incision. To oppose the operation on purely theoretical grounds, and conclude that it is a grave and serious operation, is, I believe, contrary to the facts in the published cases.

In the article alluded to in the commencement of the paper, we find a table, by a London writer, of thirty-four cases of empyema, treated surgically, occurring in children of from one to ten years of age. Of the number seventeen recovered after a short section of one rib had been made. The average time of recovery was seven weeks.

Dr. Garnett reasons that had these cases been treated with the trocar and drainage-tube they would in all probability have recovered equally as well. Here is what he says against exsection: "When we consider the fact that the bony fabric of the chest is still in a condition of progressive development, the rapid and excessive deposit of callus following the solution of continuity of a rib protruding into the cavity of the chest, irritating the lung, the inevitable disparity and loss of symmetry in expansion of the two sides of the thorax, the growing condition of the child, which necessarily adapts itself to the physical results of the traumatic interference, establishing often a permanent deformity, associated at times with more or less spondylitis, and greatly restricted respiratory capacity of the chest, the pouch of periosteum from which the excised piece of rib has been dissected, the protracted healing and long-continued dressing of the open wound, augmenting the chances of septicæmia, and exhausting the vital energies and recuperative resources of the little sufferer, we should hesitate before resorting to so grave a surgical procedure when a more simple and conservative one can be safely and successfully practiced."

Such conclusions, I think, should not go unchallenged. One does not find them from a careful study of these cases. The simple questions that I should like to invite your discussion upon is: Is subperiosteal cutting away a short segment of one rib with modern surgical treatment liable to be followed by such grave consequences as the writer referred to would have us to believe? Free drainage in abscess cavities is the imperative rule at the present day. This is best obtained by making a patulous opening larger than is possible to make between the ribs. In my first case the rib has completely re-formed. In the second it is growing again. In neither case did callus injure the lung. In both cases lung expansion and chest motion and shape are perfect. Septicæmia, present in both cases, was quickly cut short by free irrigation, only possible through a large opening. I would, therefore, conclude that in these two cases section of the rib at least added nothing to the gravity of either case.

